# Navigating relapsed hepatoblastoma: Predictive factors and surgical treatment strategy

**DOI:** 10.1002/cam4.6705

**Published:** 2023-11-14

**Authors:** Andres F. Espinoza, Kalyani R. Patel, Priya B. Shetty, Richard S. Whitlock, Pavel Sumazin, Xinjian Yu, Stephen F. Sarabia, Martin Urbicain, Andras Heczey, Prakash Masand, Sarah E. Woodfield, Dolores H. López‐Terrada, Sanjeev A. Vasudevan

**Affiliations:** ^1^ Division of Pediatric Surgery, Michael E. DeBakey Department of Surgery, Texas Children's Surgical Oncology Program and Liver Tumor Program, Dan L. Duncan Cancer Center Baylor College of Medicine Houston Texas USA; ^2^ Department of Pathology and Immunology Texas Children's Hospital, Baylor College of Medicine Houston Texas USA; ^3^ Department of Pediatric Hematology and Oncology Texas Children's Hospital, Baylor College of Medicine Houston Texas USA; ^4^ Singleton Department of Pediatric Radiology Texas Children's Hospital, Baylor College of Medicine Houston Texas USA

**Keywords:** hepatoblastoma, patient‐derived xenograft, relapse

## Abstract

**Objective:**

Hepatoblastoma (HB) is the most common primary hepatic malignancy in childhood. Relapse occurs in more than 50% of high‐risk patients with a high mortality due to ineffective salvage therapies. The purpose of this study is to identify risk factors for relapsed HB and predictors of survival in a single tertiary referral center.

**Methods:**

A retrospective chart review showed 129 surgically treated HB patients from October 2004 to July 2020. Of the cohort, 22 patients presented with relapsed HB. Relapse was defined as re‐appearance of malignancy after 4 weeks of normalized AFP and disappearance of all tumors on imaging.

**Results:**

Patients with relapsed HB had a 5‐year overall survival (OS) of 45.4% compared to 93.1% in those without relapse (*p* = 0.001). When comparing PRETEXT IV, microvascular invasion, metastatic disease, and age on multivariate logistic regression, only PRETEXT IV was an independent risk factor for relapsed HB with an OR of 2.39 (95% CI: 1.16–4.96; *p* = 0.019). Mixed epithelial and mesenchymal HB (12/19, 63.2%) was the most common histology of primary tumors while pure epithelial HB (13/15, 86.6%) was the most common relapsed histology. Combination of surgical and medical therapy for relapsed disease was predictive of survival with an HR of 16.3 (95% CI: 1.783–149.091; *p* = 0.013) compared to only chemotherapy.

**Conclusions:**

This study demonstrates that PRETEXT IV staging is an independent predictor of relapsed disease. The most common relapsed histology was epithelial, suggesting a potential selection or resistance of this component. Surgical resection is a critical component of multimodal therapy for relapsed HB.

## Graphical abstract

We demonstrate that adding surgery to relapsed HB care results in significant improvement in survival. Pretreatment extent of disease (PRETEXT) IV appears to be an independent risk factor for relapsed HB.

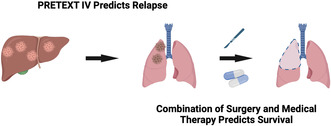

## 1 INTRODUCTION

Hepatoblastoma (HB) remains the most common liver malignancy in children.^1^, ^2^ The overall survival (OS) for these patients has significantly improved with the implementation of platinum‐based chemotherapy regimens and surgical advancements.^3^, ^4^ However, relapsed disease remains a major cause of mortality.^4^ Relapsed HBs generally pose a two‐fold hurdle as they tend not to respond well to standard chemotherapy, and can also be difficult to surgically resect, primarily due to their location.^4^ In 2013, Semeroaro et al. and the Childhood Liver Tumors Strategy Group (SIOPEL) studied a total of 59 HB patients treated in Europe that relapsed.^4^ The study showed that retrospectively, over a follow‐up period of 7 years, OS was less than 50%, and event‐free survival (EFS) to recurrence, progression, or death was less than 40%.^4^ This is the largest known published article from Europe, studying patient characteristics and outcomes associated with relapsed HB.

Advances have been made recently, with studies showing irinotecan to be an effective adjunct for relapsed disease.^5^, ^6^ In addition, recent studies have validated that the combination of chemotherapy and surgical treatment is critical for long‐term survival in newly diagnosed HB.^7^ Our group has contributed to the advancement by previously publishing the effectiveness of surgical resection of relapsed HB in the lung with a follow‐up of 18.5 months.^8^ To note, there are no studies in North America that have focused on long‐term outcomes and risk factors associated with relapsed HB to date.

Major strides have been made to risk stratify HB patients including those obtained from the Children's Hepatic tumors International Collaboration (CHIC) database analysis, identifying, and recognizing HCN‐NOS subtype and pathologic microvascular invasion being associated with higher risk disease.^9^, ^10^, ^11^ Nonetheless, there remains a need to identify factors that are highly predictive of relapse, to provide a personalized treatment regimen including intense chemotherapy or closer monitoring that could improve survival.^4^, ^12^ Many different groups have attempted to indirectly address this issue. The largest contribution came from CHIC group which proposed the current standard for risk stratification in HB.^9^ This study showed that risk factors, or “annotation factors,” that influenced the EFS included pretreatment extent of disease (PRETEXT), older age, elevated alpha‐fetoprotein (AFP), vascular involvement, multifocal disease, extrahepatic extension, tumor rupture, and metastasis.^9^ While CHIC focused on multiple endpoints that lead to EFS, little is known about predictive and prognostic factors of patients with relapsed disease. In this study, we sought to better understand predictive factors that can help practitioners better identify HB patients with the highest risk of relapse.

## 2 MATERIALS AND METHODS

### 2.1 Patient population

This retrospective review was approved by the Institutional Review Board of Baylor College of Medicine/Texas Children's Hospital (IRB #H‐50650). HB patients under the age of 18 who underwent surgical treatment at Texas Children's Hospital from January 2004 to January 2020 were included. All patient records were reviewed and patient's characteristics including gender, race, past medical history, age, treatment strategy chemotherapy, radiation, type of resection, and overall outcome over an average of 5 years were obtained. Patients initially treated at another center and who received relapsed care at Texas Children's Hospital were also included. Therapies for all these patients were evaluated by a multidisciplinary team including pathologists, medical oncologists, radiologist, and surgeons. Relapsed disease was defined as re‐appearance of HB after a 4‐week period of normal AFP and with no evidence of residual tumor, based on the same criteria that SIOPEL had previously used.^4^ The OS of patients who relapsed and the ones that did not relapse from the time of initial resection was evaluated.

### 2.2 Pathology and Radiology review protocols

All available pathology material including pathology reports, histology slides, digital images, and digitally scanned slides were re‐reviewed by our collaborating pathologists (KP/DLT). Based on the histomorphology findings, tumors were sub‐divided into epithelial (those with fetal and/or embryonal components), mesenchymal (defined by the presence of cartilage, spindle cells, and osseous tissue), teratoid (presence of neuroepithelial/glial, mucinous glands, melanin, and squamous epithelium), and blastemal (undifferentiated primitive cells). Pure fetal tumors were defined as those with only mitotically inactive (<2/10 high power field) fetal component, and absence of all other histologic differentiation. Areas with cellular pleomorphism in the form of large cells with large, irregular, hyperchromatic nuclei, coarse chromatin, prominent nucleoli, intracellular inclusions accompanied with macro trabecular architecture and brisk mitoses including atypical forms, were deemed as pleomorphic or hepatocellular carcinoma (HCN‐NOS). Percent necrosis of primary and relapsed tumors were separated into groups of less or more than 30% when evaluating prognosis.^13^ Imaging reports were reviewed to determine the extent of disease using PRETEXT staging, with the aid of an experienced radiologist (PM).

### 2.3 Statistical analysis

Patient demographics and clinical characteristics were reported using interquartile ranges (IQR) and medians. SPSS 28.0.1 was used to perform chi‐squared analysis, Mann–Whitney *U*, and multivariate analysis. Kaplan–Meier curves were used to illustrate OS and log‐rank test were utilized to test survival analysis. Survival and recurrence were calculated from time of initial resection to date of last follow‐up or date of event.

## 3 RESULTS

**FIGURE 1:**
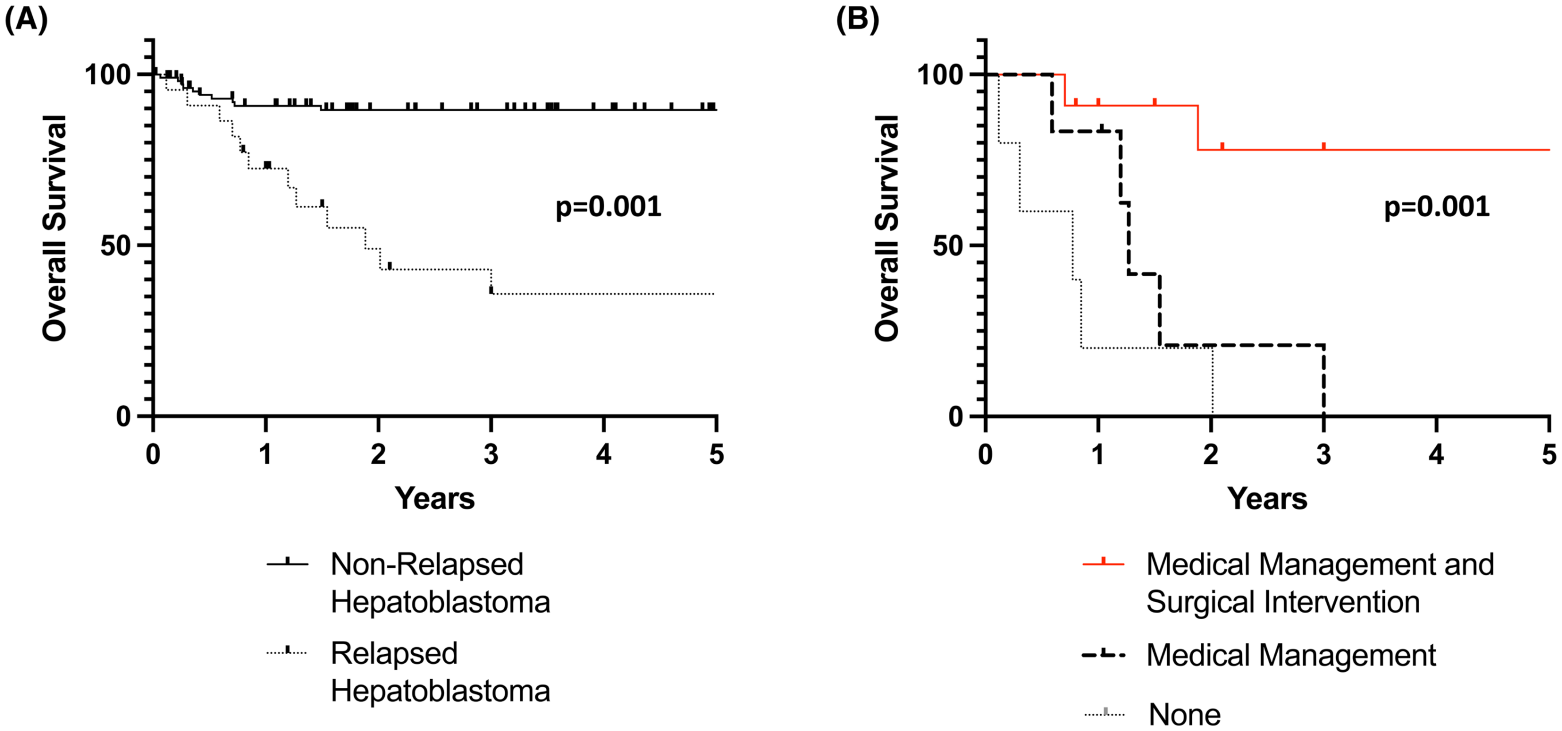
(A) The overall survival of relapsed patients versus those without relapse is plotted over a 5‐year interval since resection. There was a 93.1% overall survival in the non‐relapsed patients versus 45.4% survival in the relapsed patients (*p* = 0.001). (B) The overall survival of the relapsed patients that underwent medical management, medical management, and surgical intervention, or none over a 5‐year interval since resection. There was an 80% overall survival in the medical management and surgically intervened patients versus 0.0% in the medically managed cohort (*p* = 0.001) at 5‐year interval. The patients with no management, due to progression of disease, all died of disease. To note, one patient in the medically managed cohort was alive but was lost to follow‐up after the 1.1 year mark.

Of the 129 patients diagnosed with HB that received care at our center, 22 were found to have relapsed disease. Of these, seven patients were initially treated with transplant, while 15 patients were treated with resection. The cohort of patients that relapsed had an OS of 45.4% over a follow‐up period of 5 years. In contrast, the patients that did not relapse had an OS of 93.1% over the same time interval (*p* = 0.001) (Figure 1). When comparing the demographics of the patients (Table S1), age at initial diagnosis (33.4 months for relapsed vs. 17 months for non‐relapsed disease) was found to be statistically significant (*p* = 0.002).

Of the 22 patients with relapsed disease, histologic material was available for 19 primary tumors (86.4%). Relapse tumors were sampled/resected in 15 patients (68.2%), all of which were available for pathology review. A total of 12 paired samples were available for histopathologic comparison. Tumors were sub‐divided into three primary subtypes (mutually exclusive) with or without additional components (Table S2). A given tumor could have none, one, or more than one additional component as shown in Table S3. All primary tumors were resected/explanted after chemotherapy. Based on the older age (>5 years) and presence of pleomorphic areas in both pretreatment biopsy and post‐treatment resection samples, two patients were diagnosed as hepatocellular neoplasm, not otherwise specified (HCN‐NOS). The most common histology in the primary tumor was mixed epithelial and mesenchymal HB (12/19, 63.2%), followed by pure epithelial (7/19, 36.8%). Pure fetal histology was not seen in any primary tumor. The most common histology in the relapse sample was pure epithelial HB with a variable combination of fetal and embryonal components (13/15, 86.6%) (Figure S1), followed by mixed epithelial mesenchymal (2/15, 13.33%). Pure fetal or teratoid histology was not seen in any of the sampled relapse tumors. To note, five patients had a minor blastemal component in the primary (typically 10%–30% areas). Of the three patients with relapse specimens, none showed evidence of blastemal component. Of the seven patients with pleomorphic areas in their primary tumors, five did not have their relapse tumors biopsied or resected. Of the remaining two, one showed relapse with embryonal HB (no pleomorphism) and the other showed a similar pleomorphic component in the relapse that was GPC‐3 negative. Interestingly, two of the three pleomorphic relapse tumor specimens were de novo, given that their primary tumors had not shown any pleomorphic component.

Of the 20 patients that had CHIC‐HS risk available on the electronic medical record (EMR), 8 (40%) were noted to be high risk, 9 (45%) intermediate, and 3 (15%) were low risk (Table 1. The variation between risk category, time from neoadjuvant chemotherapy to surgery, and surgical approach for each patient's primary tumor, presence of negative margins, and percent necrosis at time of primary resection were not predictive of survival in our cohort (Tables 2 and 3). The level of necrosis or response to therapy based of Response Evaluation Criteria of Solid Tumors (RECIST) for the relapsed tumors was not predictive of survival as well.^14^ In addition, treatment with medical management only was not predictive of survival (OR of 0.1 [95% CI: 0.015–1.653]; *p* = 0.12). Patients that were treated with multimodal therapy including surgical resection of the relapse disease had a 16.3 (95% CI: 1.783–149.091; *p* = 0.013) hazard ratio of survival compared to only chemotherapy, as shown in Table 3. When analyzing survival of the relapse cohort according to treatment modality, the patients treated with surgery and chemotherapy had a significantly improved 5‐year OS of 80% and 5‐year EFS of 72.7% compared to 0% for chemotherapy only and 0% for no treatment (*p* = 0.001, Figure 1; Figure S3). Furthermore, we compared prognostic factors and outcomes between the types of primary local control surgery including partial hepatectomy and liver transplantation (Table S4). Of known high‐risk disease characteristics, V/P+ and metastatic disease did not prove to be a predictive factor for relapsed disease, with 78% of our metastatic cohort and 76.7% of our V/P+ cohort not relapsing. We found that despite patients that underwent resection more often had metastasis at diagnosis, microvascular invasion, and PRETEXT IV disease, the OS was similar for both (Figure S2). The median follow‐up for the transplant cohort was 2.85 years (IQR 2.3–3.39 years) while the partial hepatectomy cohort was 2.1 years (IQR 1–8.9 years).

**TABLE 1:** Patient's primary tumor, PRETEXT, annotation factors, age of diagnosis, risk group, treatment course, and outcome of the relapsed hepatoblastoma patients.

ID	PRETEXT	Annotation factors	Age of diagnosis (years)	Risk group	Chemotherapy neoadjuvant/Adjuvant	Surgical approach	Outcome
1	IV	P	1.7	IR	VI (4)/Dox (5)	Standard Hepatectomy	DOD
2	III	None	11.3	HR	C5D (4)/C5D (2)	Extended Hepatectomy	DOD
3	IV	V, P	8	HR	C5VD (4)/C5VD (2)	Extended Hepatectomy	DOD
4	IV	P, M	6.4	IR	Cis and Dox (3)/None	OLT	Alive
5	IV	V, P	1.2	IR	C5VD (3)/None	OLT	Alive
6	IV	P	9.9	IR	C5VD (3)/ICE (2), 3 months of sorafenib	OLT	DOD
7	IV	None	1.5	IR	C5VD (6)/None	OLT	Alive
8	IV	V, P	1.7	IR	C5VD (2), ICE (1), VI (1)/PO irinotecan	OLT	DOD
9	III	None	0.7	IR	NA^a^/C5V (2)	Standard Hepatectomy	DOD
10	III	None	4.9	IR	C5V (4)/Ifos and dox (3)	Extended Hepatectomy	DOD
11	III	V, M	2.3	HR	C5V (1)/None	Standard Hepatectomy	Alive
12	IV	V, P, M	2.7	HR	C5V (5)/Cis and Dox (2)	Extended Hepatectomy	DOD
13	IV	M	2.3	HR	C5VD (2)/C5VD (3)	Standard Hepatectomy	DOD
14	III	M	2.7	HR	C5VD (2)/C5VD (3)	Extended Hepatectomy	Alive
15	IV	None	2.9	IR	Cis (4)/None	OLT	DOD
16	IV	P	1.9	HR	VIT (4)/C5VD (6)	Standard Hepatectomy	Alive
17	NA	None	2.5	LR	None/C5V (2)	Standard Hepatectomy	Alive
18	IV	None	4.9	NA	NA^a^/NA^a^	OLT	DOD
19	NA	M	7.7	NA	C5V/C5V^a^	Standard Hepatectomy	Alive
20	III	None	0.6	LR	None/C5VD (4)	Extended Hepatectomy	DOD
21	II	None	0.6	LR	None/Cis (2)	Standard Hepatectomy	Alive
22	II	V, M	3.8	HR	C5VD (5), VI (1)/ICE (3)	Standard Hepatectomy	Alive

Abbreviations: C5D, cisplatin/5‐FU/doxorubicin; C5V, cisplatin/5‐FU/vincristine; C5VD, cisplatin/5‐FU/vincristine/doxorubicin; carbo/dox, carboplatin/doxorubicin; Cis, cisplatin; DOD, dead of disease; Dox, doxorubicin; ICE, Ifosfamide/carboplatin/etoposide; Ifos/dox, ifosfamide/doxorubicin; IT, Irinotecan/Temozolomide; M, metastatic disease present at diagnosis; NA, not available; P, involvement of portal vein—both left and right portal vein, or portal bifurcation, or both; TIT, Temsirolimus/Irinotecan/Temozolomide; V, involvement of vena cava—all three hepatic veins or the intrahepatic inferior vena cava, or both; VI, vincristine/irinotecan; VIT, vincristine/irinotecan/temozolomide.

^a^ Patient received chemotherapy but the amount of cycles and the exact therapy scheme was not available.

**TABLE 2:** Patient's relapse treatment scheme, response to therapy through RECIST/percent necrosis, and outcome of the relapsed hepatoblastoma patients.

ID	Age of relapse (years)	Time to relapse (Days)	Relapse medical therapy	Response to medical therapy (RECIST criteria)	Location/Type of relapse surgery	Percent viability (number of nodules)	AFP at recurrence	Outcome
1	3.2	142	None	NA	None	NA	207,200	DOD
2	12.1	91	VI (NA)	PD	None	NA	45,000	DOD
3	9.3	218	None	NA	None	NA	22	DOD
4	6.9	123	NA	NA	None	NA	404	Alive
5	2.1	294	4 cycles of VI (4), irinotecan (1)	NA	Left lung/UT	100% (1)	105	Alive
6	1.2	535	Lenvatinib (5 months), TARE	PD	Right Lung/UT	100% (13)	214	DOD
7	3.1	507	ICE (6), PO irinotecan (10)	PD	Segment 8/PH	98%	44	Alive
8	1.8	29	None	NA	None	NA	77,000	DOD
9	1.0	230	None	NA	None	NA	24	DOD
10	5.5	182	VIT (2), cyclo (2)	PD	None	NA	2422.8	DOD
11	3.2	224	First relapse—Ifos/dox (1), VI (10), TIT (2)	First relapse—SD	First relapse—left lung/UT	100% (1)	34.9	Alive
			Second relapse—IT (6)	Second relapse—NA	Second relapse—right lung/UT	100% (1)		
12	3.7	202	VI (1), ICE (1)	PD	None	NA	22	DOD
13	3.0	189	First relapse—VI (2), ICE (1)	First relapse—NA	First relapse—right lung/left lung/BT	50% (13)/5% (6)	719	DOD
			Second relapse—XRT	Second relapse—SD	Second relapse—right lung/UT	100% (1)		
			Third relapse—Sirolimus PO, cis (1), carbo/dox (1)	Third relapse—PD	Third relapse—right lung/UT	100% (1)		
14	3.4	230	VI (4)	NA	Right lung/UT	100% (1)	84	Alive
15	3.4	98	None	NA	None	NA	215	DOD
16	3.5	442	ICE (4)	SD	Segment 8/PH	80%–85% (1)	964	Alive
17	2.9	150	First relapse—C5VD (6)	First relapse—NA	First relapse—Left lung/UT	100% (3)	257	Alive
			Second relapse—ICE (2), VI (1), CAR T‐Cell (1)	Second relapse—NA	Second relapse—Bilateral lungs (staged)/BT	100% (2)		
			Third relapse—None	Third relapse—NA	Third relapse—Brain/C	100% (1)		
18	6.4	436	NA^a^	NA	NA^a^	NA	NA	DOD
19	4.3	193	Ifos/Dox (NA)	SD	Segment 8/OLT	75%–80% (1)	4107	Alive
20	2.7	358	VI (1)	PD	None	NA	908	DOD
21	0.9	31	VI (2)	PR	Bilateral lungs/BT	100% (13)	NA	Alive
22	7.7	90	NA^a^	PD	Left lung/UT	100% (1)	108	Alive

Abbreviations: AFP, alpha‐fetoprotein; BT, bilateral thoracotomy; C, craniotomy; C5D, cisplatin/5‐FU/doxorubicin; C5V, cisplatin/5‐FU/vincristine; C5VD, cisplatin/5‐FU/vincristine/doxorubicin; carbo/dox, carboplatin/doxorubicin; Cis, cisplatin; DOD, dead of disease; Dox, doxorubicin; ICE, Ifosfamide/carboplatin/etoposide; Ifos/dox, ifosfamide/doxorubicin; IT, Irinotecan/Temozolomide; NA, not available; PD, progressive disease; PH, partial hepatectomy; PR, partial response; SD, stable disease; TIT, Temsirolimus/Irinotecan/Temozolomide; UT, unilateral thoracotomy; VI, vincristine/irinotecan; VIT, vincristine/irinotecan/temozolomide.

^a^ Patient data were not available for review.

**TABLE 3:** Log‐rank analysis evaluating factors associated with overall survival within the relapsed hepatoblastoma cohort.

	Hazard ratio (95% CI)	*p*
Risk category		
High	Reference Group	0.76
Intermediate	1.3 (0.196–9.083)	0.23
Low	5.3 (0.343–82.831)	
Time from neoadjuvant chemotherapy to surgical resection		
>20 days	Reference Group	0.77
<20 days	1.3 (0.190–9.311)	
Surgery for primary disease		
OLT	Reference Group	0.32
Extended hepatectomy	0.26 (0.019–3.653)	0.79
Standard hepatectomy	1.3 (0.149–11.929)	
Percent necrosis of primary tumor		
≥30%	Reference Group	0.74
<30%	0.6 (0.040–9.653)	
Margin status of primary tumor		
Negative	Reference Group	0.64
Positive	1.6 (0.194–14.266)	
Management for relapsed disease		
Medical management	Reference Group	0.69
No management (Rapid Progression of Disease)	0.7 (0.184–3.115)	0.013
Medical management and surgical intervention	16.3 (1.783–149.091)	
Percent necrosis of relapsed tumor		
≥30%	Reference Group	0.53
<30%	0.2 (0.004–16.864)	
Relapsed tumor response (RECIST Criteria)^a^		
Progressive disease	Reference Group	
Stable disease	13.0 (0.447–377.493)	0.13
Partial response	7.8 (0.231–262.827)	0.25

^a^ RECIST was based on the last relapse the patient experienced.

We then compared PRETEXT IV, microvascular invasion, metastasis at diagnosis, and age of diagnosis of the relapsed HB cohort by univariate analysis. As shown in Table 4, only microvascular invasion and PRETEXT IV were noted to have a statistically significant odds ratio of 3.42 (95% CI: 1.07–10.87; *p* = 0.038) and 2.34 (95% CI: 1.16–4.71; *p* = 0.017), respectively. On multivariate analysis, PRETEXT IV was an independent risk factor for relapsed HB with OR of 2.39 (95% CI: 1.16–4.96; *p* = 0.019) when adjusting for age, metastasis at time of diagnosis, and microvascular invasion at time of diagnosis, as shown in Table 4. When looking at the risk factors that were more prominent in relapsed patients that survived, the strongest predictive factor was receiving multimodal therapy including surgery for relapse (Table S5).

**TABLE 4:** Multivariate regression analysis showing univariate (crude odds ratio) and multivariate (adjusted odds ratio) results of risk factors associated with relapsed hepatoblastoma.

	Crude odds ratio (95% CI)	*p*	Adjusted odds ratio (95% CI)	*p*
Age (months)	1.0 (1.0–1.001)	0.022	1.0 (1.0–1.001)	0.078
PRETEXT IV	2.34 (1.16–4.71)	0.017	2.39 (1.16–4.96)	0.019
Metastatic disease at diagnosis	1.53 (0.56–4.17)	0.405	1.21 (0.35–4.22)	0.769
Microvascular Invasion	3.42 (1.07–10.87)	0.038	1.98 (0.48–8.09)	0.348

## 4 DISCUSSION

Relapsed disease continues to be a leading cause of death in patients diagnosed with HB.^4^ Despite improvements in medical therapy and surgical advancements, less than 50% of the relapsed patients in our cohort and other studies have been shown to survive.^4^ This highlights the need to further understanding, not only the biology of relapsed HB, but also how to better risk stratify these patients, provide appropriate therapy, and monitor disease. This would allow for different therapeutic approach selection for the initial lesion, the relapsed tumor, and closer monitoring when warranted. In our cohort, there appears to be a clear association between microvascular invasion, and older age of diagnosis with a higher incidence of relapsed disease. The strongest predictor for relapsed disease appears to be disease in all sections of the liver (PRETEXT IV), with patients having 2.39 times higher chance of experiencing relapse when accounting for the previously mentioned factors. When evaluating the treatment schemes of our relapsed cohort, the only management that appeared to be predictive of survival was surgical resection combined with chemotherapy for the relapsed tumors. This emphasizes the need for aggressive multimodal approach to help salvage relapsed HB.

We found that within our relapsed cohort, the pure epithelial relapsed tumors showed embryonal component more frequently and more widely than the fetal component. Despite this, HBs with epithelial and mesenchymal components were noted to be the most common primary tumor histology (63.2%) (Table S2). Similarly, there were no teratoid components identified in the relapsed tumors in our series as, except for immature neuroepithelium, heterologous tissues that define teratoid HBs are typically benign in appearance. This supports the concept that embryonal histology may be more often associated with more aggressive biology and worse prognosis.^15^ Given this, further efforts to evaluate the utilization of embryonal histology as a clinical risk factor may be warranted.

PRETEXT scoring system was initially published in 2007 by the SIOPEL group to aid physicians in categorizing HB involvement the liver.^12^, ^16^ The PRETEXT scoring system is currently the standard imaging staging method for HB dividing the liver into four separate sections with 6/7, 5/8, 4a/b, and 2/3.^12^, ^16^ PRETEXT IV is described as having no uninvolved sections, in other words, disease in all four sections of the liver.^16^ The PRETEXT scoring system has been used in many studies to help risk stratify HB patients and create treatment schemes.^9^ One of the largest and most cited studies was published by Meyers et al. and the CHIC group in 2017, which served to advance knowledge on risk stratification and predicting outcomes for HB. This study showed that only PRETEXT IV patients that had no metastasis at diagnosis, were younger than 3 years of age, had AFP greater than 100 ng/mL and were did not have any annotation factors, were considered intermediate risk.^9^ All other PRETEXT IV groups were found to be high risk and thus would receive more aggressive therapy.^9^ Our study supports the concept that PRETEXT IV is predictive of high‐risk disease and warrants aggressive chemotherapy and surgical treatment. When looking at our relapsed cohort, 4 of 6 PRETEXT IV patients who were placed into the intermediate risk treatment arm succumbed to their relapsed disease, supporting our multivariate regression analysis showing that PRETEXT IV may warrant re‐evaluation for high‐risk disease. Our study suggests that all HB patients with PRETEXT IV disease may benefit from being categorized as “high risk,” given the independent risk resulting in relapsed disease.

Interestingly, within our cohort we had three patients that relapsed despite being categorized initially as low risk. All three low risk patients underwent upfront resection and were treated with adjuvant chemotherapy as shown in Table 2 (Patient #17, #20, #21). Two patients were able to be salvaged with both combination of surgery and intense chemotherapy (Patient #17, #21). Patient #17 was salvaged despite having multiple relapses that required three different chemotherapy schemes and surgical interventions including a craniotomy while patient #21 had bilateral thoracotomies along with two cycles of vincristine/irinotecan (Table 3). First, these patients emphasize the importance of aggressive surgical approach required to treat relapsed HB, despite multiple relapses. In addition, we show that a certain cohort of patients classified by the current risk schema as “low risk” HB tumors may in fact have biology more consistent with high‐risk disease. We hypothesize that this may be due to the heterogenous nature of HB, circulating tumor cells (CTCs), or dormant metastatic deposits that evade standard chemotherapy and even detection.^1^, ^17^ This finding emphasizes the urgent need for a more biology‐based risk stratification to take these factors into account when deciding on adjuvant therapies.

The mainstay therapy for HB relies on combination of chemotherapy and aggressive surgical resection.^18^ While surgical and medical advances have allowed us to improve OS for HB patients, these patients rely on a personalized surgical approach and perioperative chemotherapy.^18^, ^19^ Despite this, there are currently no guidelines on how to manage relapsed HB, thus relying on single‐center experience to guide management.^18^ Our manuscript presents that the utilization of both medical and surgical management for relapse disease is critical to salvage these patients. When evaluating relapsed patients that were salvaged with multimodal therapy, we could not find any predictive RECIST response or percent necrosis on final pathology. We hypothesize that these patients were salvaged due to the macroscopic disease that may be less sensitive to chemotherapy being surgically removed while the chemotherapy eradicates the microscopic residual disease.^13^, ^18^ As shown in Table 2, aggressive surgical approaches proved to be effective in relapsed disease, at times requiring multiple resections or staged approaches. To note, two of the three patients with multiple relapses, Patient #11 and patient #17, were salvaged with multimodal treatment strategies including aggressive surgical resection of the relapse lesions. Likewise, patient #6 and patient #21 had each 13 individual nodules of HB removed and both patients were salvaged. Interestingly, we noticed that the patients that underwent upfront resection of their relapsed disease followed by adjuvant chemotherapy had a slightly lower OS (40%) compared to those that underwent neoadjuvant chemotherapy before metastectomy (60%). Given the similar OS, we could not discern the difference in outcomes between the patients that underwent liver transplantation versus partial hepatectomy. Given this, we would recommend the current transplantation criteria of PRETEXT III V/P+ and PRETEXT IV, while considering extreme resection for metastatic disease. While in our cohort only one patient underwent liver transplantation for relapsed disease, our institution has not historically offered transplantation for relapsed disease. This notion is currently being challenged given recent studies from Boster et al. which have shown the “salvage transplantation” for relapsed HB has survival rates that now approach 62% compared to the previously reported 40%.^20^, ^21^

This study has limitations that should be noted. The study is a retrospective analysis, despite it presenting one of the largest cohorts of relapsed HB in the United States. We recognize that to further validate the risk factors we studied in predicting relapse, larger multicenter studies are warranted. A limitation in our study is the variability in chemotherapy regimens for both primary and relapsed tumors which presents a challenge to evaluate the response of specific pathological components of the tumors. To note, of the seven patients with pleomorphic histology of the primary tumor, two patient's relapsed tumors were available to be reviewed and thus pleomorphic histology as a risk factor may not be fully evaluated. In addition, the heterogeneity of the chemotherapy limits our ability to discuss recommendations concerning medical therapy at the time of relapse. While other groups have shown that cisplatin‐based re‐treatment for relapsed HB is beneficial, the heterogeneity of the chemotherapy that our cohort received limits our sub analysis.^22^ Despite this, our manuscript was focused on the role of surgical management for relapsed disease, which proved to benefit the patients.

Patients with relapsed HB remain difficult to treat given limited therapeutic options and the lack of definitive risk stratification data.^1^, ^2^, ^3^, ^4^ This is secondary to the rarity of the disease and limited clinical and biological data from patients that can contribute to large data sets, such as that our center was able to provide.^1^, ^2^, ^3^ Our study shows that the addition of surgical resection to medical management plays a critical role in salvaging patients with relapsed HB. In addition, we demonstrated that PRETEXT IV stage disease alone was predictive of disease relapse for patients treated in our center. Our data confirm that relapsed patients have overall low salvage and survival rates,^1^, ^2^, ^3^ supporting previous studies showing that PRETEXT IV is a subset of patients with high‐risk disease that require intensive therapy,^9^, ^12^, ^16^, ^23^ and emphasizes the importance of pursuing surgical therapy for these patients.

## AUTHOR CONTRIBUTIONS

**Andres F. Espinoza:** Conceptualization (equal); data curation (equal); formal analysis (equal); funding acquisition (equal); investigation (equal); methodology (equal); project administration (equal). **Kalyani R. Patel:** Conceptualization (equal); data curation (equal); formal analysis (equal); funding acquisition (equal). **Priya B. Shetty:** Formal analysis (equal). **Richard S. Whitlock:** Data curation (equal). **Pavel Sumazin:** Formal analysis (equal). **Xinjian Yu:** Formal analysis (equal). **Stephen F. Sarabia:** Formal analysis (equal). **Martin Urbicain:** Formal analysis (equal). **Andras Heczey:** Investigation (equal); methodology (equal). **Prakash Masand:** Investigation (equal). **Sarah Woodfield:** Investigation (equal). **Dolores H. López‐Terrada:** Investigation (equal). **Sanjeev A. Vasudevan:** Conceptualization (equal); data curation (equal); formal analysis (equal); funding acquisition (equal); investigation (equal); methodology (equal); project administration (equal).

## CONFLICT OF INTEREST STATEMENT

The authors do not report any conflicts of interest.

## Supporting information


Figures S1–S3.

Tables S1–S5.

## Data Availability

Data sharing is not applicable to this article as no new data were created or analyzed in this study.
