# Supplementary material for: Navigating relapsed hepatoblastoma: Predictive factors and surgical treatment strategy
Source: Cancer Med. 2023 Nov 14;12(23):21270–8. doi: 10.1002/cam4.6705 (PMC10726870; doi:10.1002/cam4.6705)
Supplement: Supplementary file 1 — Figures S1–S3. [file CAM4-12-21270-s001.pdf]

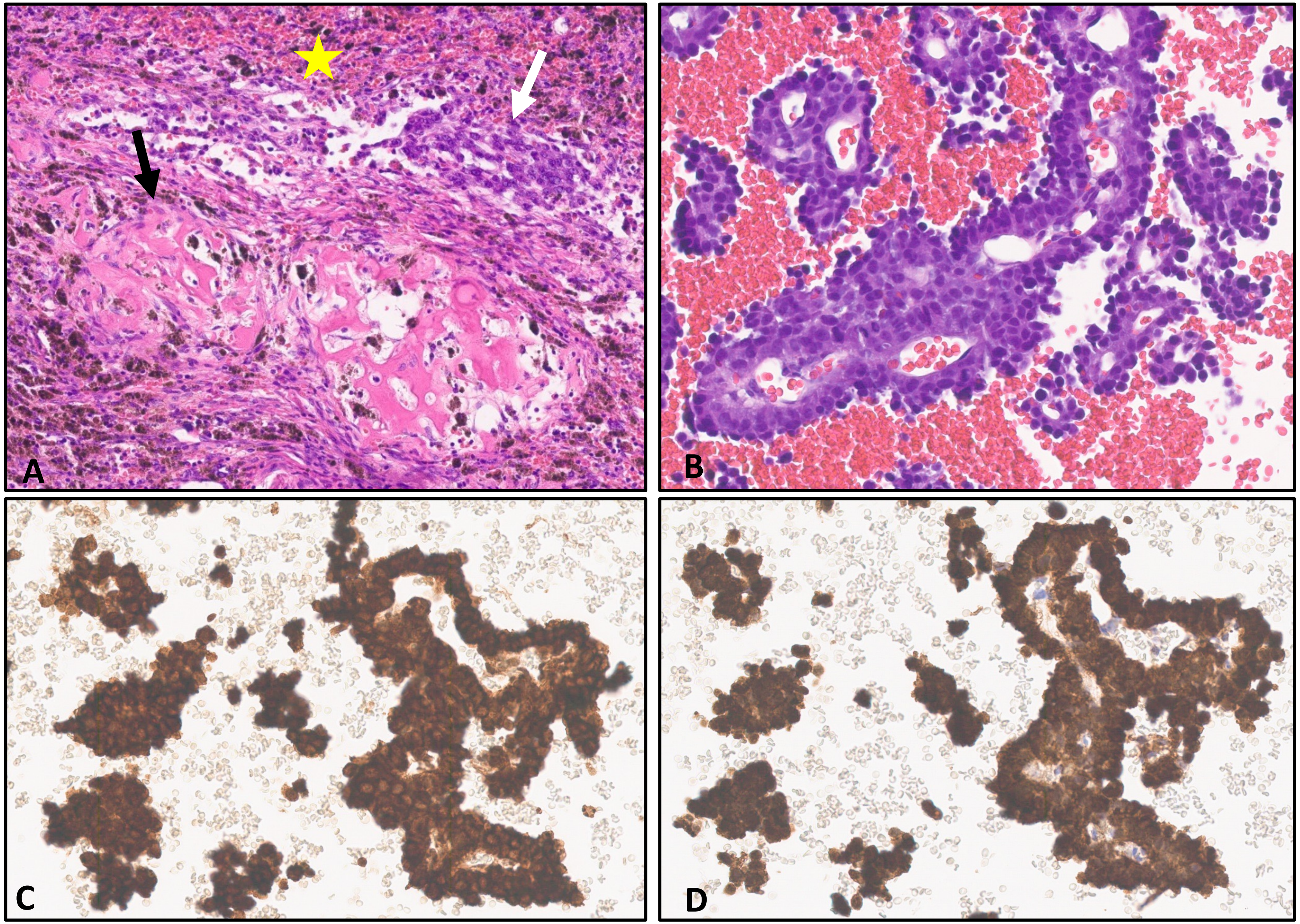

Supplementary Figure 1. A Post-therapy hepatoblastoma (10x) from a patient showing both epithelial (white arrow) and mesenchymal (black arrow) components along with therapy related hemorrhage and necrosis (yellow asterisk). Mesenchymal component was represented in the form of osseous differentiation. **B-D**: Local recurrence (20x) within 9 months showed a pure epithelial hepatoblastoma (B) with diffuse and strong glypican-3 (C) and beta catenin positivity (D).

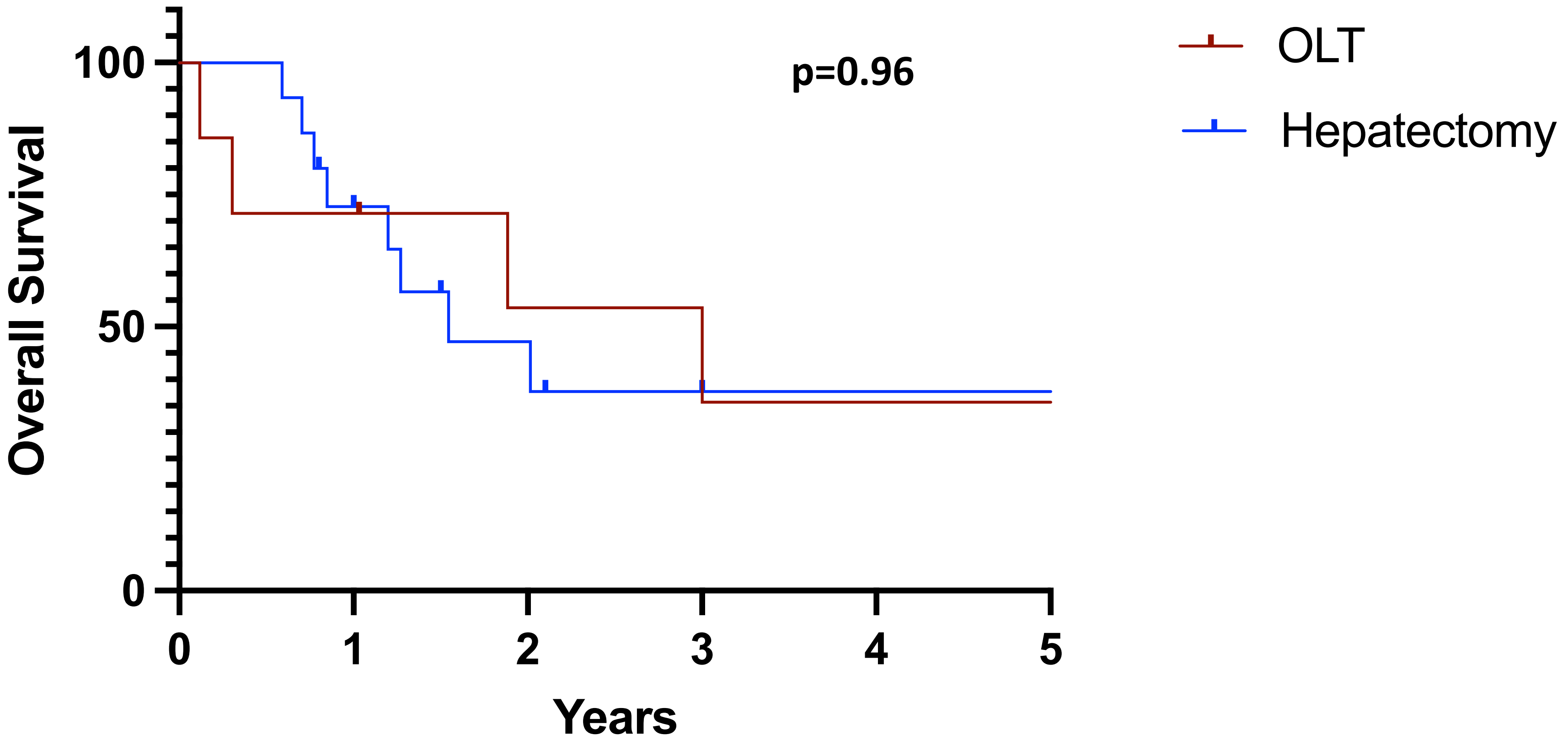

Supplementary Figure 2. The overall survival of the relapsed patients that underwent orthotopic liver transplant (OLT) versus those that underwent hepatectomy over a 5-year interval since resection. There was a 42.8% overall survival in the OLT treated patients versus 46.6% in the hepatectomy cohort (p=0.96).

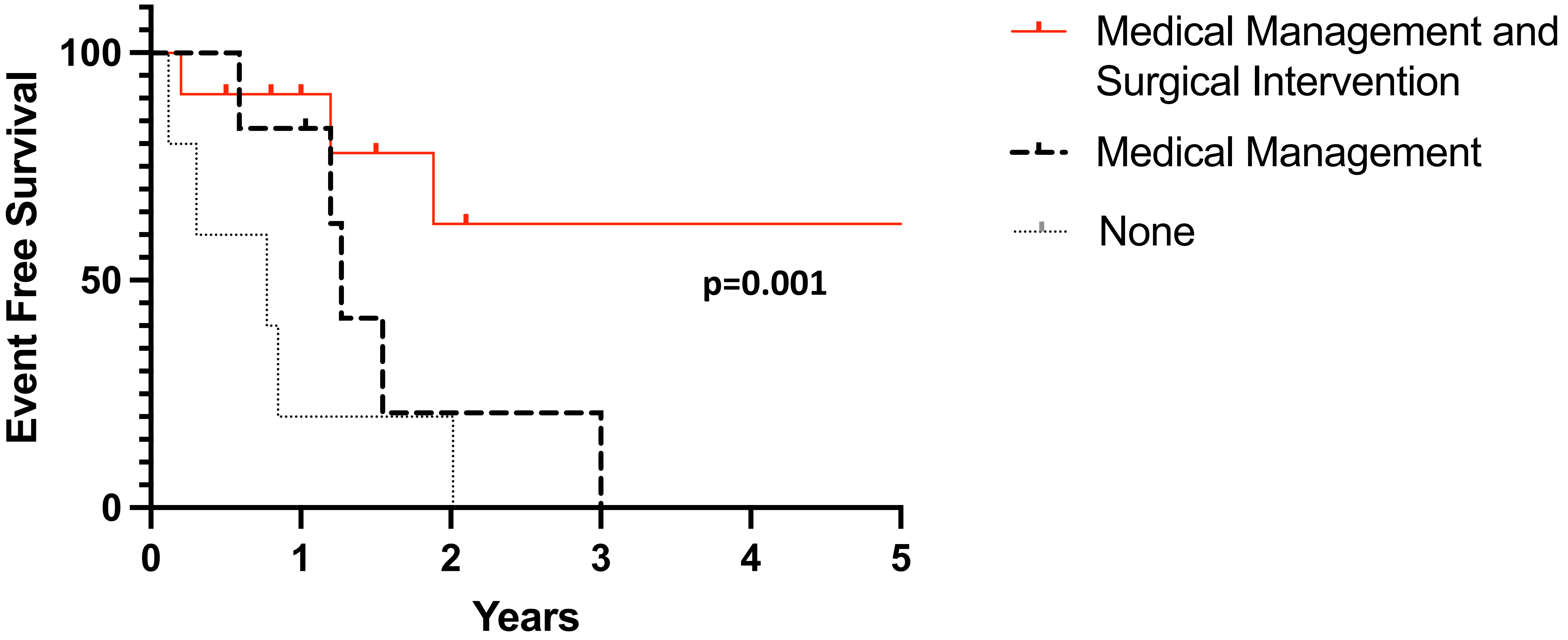

Supplementary Figure 3. The event free survival of the relapsed patients that underwent medical management, medical management and surgical intervention, or none over a 5-year interval since resection. There was an 72.7% overall survival in the medical management and surgically intervened patients versus 0.0% in the medically managed cohort (p=0.001) at 5-year interval. The patients with no management, due to progression of disease, all died of disease.

*To note one patient in the medically managed cohort was alive but was lost to follow-up after the 1.1 year mark
