# Supplementary material for: Navigating relapsed hepatoblastoma: Predictive factors and surgical treatment strategy
Source: Cancer Med. 2023 Nov 14;12(23):21270–8. doi: 10.1002/cam4.6705 (PMC10726870; doi:10.1002/cam4.6705)
Supplement: Supplementary file 2 — Tables S1–S5. [file CAM4-12-21270-s002.docx]

|  | **Relapse (n=22)** | **No Relapse (n=107)** | **p** |
| --- | --- | --- | --- |
| Age (months) | 33.4 (8-137.8) | 17 (5.6-210.8) | 0.002 |
| **Sex**  Male  Female | 16 (72.6 %)  6 (27.4%) | 69 (64.5%)  38 (35.5%) | 0.46 |
| **Race**  Caucasian  Hispanic  African American  Asian  Middle Eastern | 10 (45.5%)  12 (54.5%)  0  0  0 | 37 (35.2%)  56 (53.3%)  8 (7.6%)  3 (2.9%)  1 (1.0%) | 0.33  0.85  NA  NA  NA |
| Premature | 6 (27.3%) | 38 (35.5%) | 0.45 |
| **PRETEXT score***  I  II  III  IV | 0  2 (10.0%)  6 (30.0%)  12 (60.0%) | 8 (8.3%)  28 (29.2%)  31 (32.3%)  29 (30.2%) | NA  0.07  0.84  0.01 |
| Lung metastasis at diagnosis | 7 (31.8%) | 25 (23.4%) | 0.40 |
| V+/P+ | 10 (47.6%) | 33 (33%) | 0.20 |
| Microvascular invasion | 17 (81.0%) | 56 (55.4%) | 0.05 |

**Supplementary Table 1. Demographics and initial presentation characteristics of the relapsed and non-relapsed hepatoblastoma patients.**

**PRETEXT scores were not available for 2 relapsed patients and 11 non-relapsed patients.*

|  | **Primary Tumor Histology (n=19)** | **Relapse Tumor Histology (n=15)** |
| --- | --- | --- |
| **Primary Subtypes:**   - Pure Fetal - Epithelial Only - Epithelial and Mesenchymal | 0 (0%)  7 (36.8%)  12 (63.2%) | 0 (0%)  13 (86.6%)  2 (13.4%) |
| **Additional Components:**   - Blastemal - Pleomorphic or HCC-like - Teratoid | 7 (36.8%)  7 (36.8%)  4 (21%) | 2 (13.3%)  3 (20%)  0 (0%) |

**Supplementary Table 2. Distribution of histological subtypes found in primary and relapsed tumors.**

| **ID a** | **Primary Tumor** | **Epithelial** | **Mesenchymal** | **Teratoid** | **Embryonal** | **Fetal** | **Blastema** | **Pleomorphic** | **Post therapy necrosis** | **Margins** | **Relapsed tumor Pathology** | **Comment Primary vs Relapse** |
| --- | --- | --- | --- | --- | --- | --- | --- | --- | --- | --- | --- | --- |
| 1 | Epithelial with abundant blastema | Y | N | N | Y | Y | Y, abundant | N | NA | Negative | Mixed Fetal/Embryonal with blastema | Same as primary |
| 2 | Epithelial-mesenchymal | Y | Y | N | Y | Y | N | N | NA | NA | Mixed fetal/embryonal | No mesenchymal in relapse |
| 3 | Epithelial-mesenchymal | Y | Y | N | Y | Y | N | N | 60 | Negative | Mixed fetal/embryonal | Same as primary |
| 4 | Epithelial-mesenchymal | Y | Y | N | N | Y | N | N | 20 | Positive | Largely fetal, rare mesenchymal | Same as primary |
| 5 | Epithelial-mesenchymal | Y | Y | N | Y | Y | N | N | NA | NA | Mesenchymal (70%)>> epithelial (30%) | Mesenchymal dominant relapse |
| 6* | Pure epithelial, embryonal dominant | Y pure | N | N | Y, 90% | Y, 10% | N | N | 20 | Positive | NA | NA |
| 7 | Pure epithelial, fetal dominant | Y pure | N | N | Y, 10% | Y, 90% (mitotically inactive) | N | N | 5 | Positive | Pleomorphic 90%, embryonal 10% | Pleomorphic in relapse only |
| 8 | Epithelial-mesenchymal-teratoid | Y | Y | Y | N | Y | N | N | 20 | Negative | Pleomorphic HCC like | Pleomorphic in relapse only |
| 9 | Epithelial-mesenchymal, focal pleomorphism | Y | Y, spindle cells | N | Y, 60% | Y, 40% | N | Y | 10 | Negative | NA | NA |
| 10 | Epithelial-mesenchymal, focal pleomorphism | Y | Y, osteoid | N | N | Y | N | Y | 25 | Negative | NA | NA |
| 11 | Epithelial, pleomorphic | Y pure | N | N | N | Y | N | Y | 40 | Negative | NA | NA |
| 12 | Epithelial, pleomorphic | Y pure | N | N | Y | Y | N | Y | 30 | Positive | NA | NA |
| 13 | Epithelial-mesenchymal-pleomorphic | Y | Y | N | Y | N | N | Y, HCC like | 10 | Positive | Embryonal and pleomorphic GPC-3 negative | Similar to primary with GPC-3 negative component |
| 14 | Epithelial (embryonal dominant), with blastema | Y pure | N | N | Y, predominant | N | Y | N | NA | NA | NA | NA |
| 15 | Epithelial-mesenchymal-teratoid-Blastema | Y | Y | Y | Y | Y | Y | N | NA | NA | Mixed fetal/embryonal | Only epithelial in relapse |
| 16 | Epithelial-mesenchymal-teratoid-Blastema | Y | Y | Y | Y, 65% | Y, 30% | Y | N | 10 | Negative | Fetal and embryonal | Only epithelial in relapse |
| 17 | Epithelial-mesenchymal-teratoid-Blastema- pleomorphic | Y | Y | Y | Y | Y | Y | Y | 10 | Positive | Embryonal | Only epithelial in relapse |
| 18 | Epithelial-mesenchymal, abundant blastema | Y | Y (some striated, spindle cells) | N | Y | N | Y, abundant | N | NA | NA | Myxoid/small cell (blastema) | NA |
| 19* | Epithelial, blastema, pleomorphic | Y pure | N | N | Y, 10% | Y | Y, 10% | Y | NA | Negative | NA | NA |
| 20 | NA | NA | NA | NA | NA | NA | NA | NA | NA | NA | Microscopic foci - epithelial only | NA |
| 21 | NA | NA | NA | NA | NA | NA | NA | NA | NA | NA | Epithelial with anaplasia | Relapse is morphologically like the primary per report |
| 22 | NA | NA | NA | NA | NA | NA | NA | NA | NA | NA | Mixed fetal/embryonal | NA |

**Supplementary Table 3. Comparative histologic assessment of primary and relapse tumors.**

**=Patient had resection of relapsed tumor but tissue was not available for evaluation, NA=NA, HCC=hepatocellular carcinoma*

|  | **Transplant (n=7)** | **Resection (n=15)** | **p** |
| --- | --- | --- | --- |
| **Age (months)** | 34.7 (17.8-77.4) | 32.5 (22.4-67.7) | 0.971 |
| **PRETEXT IV** | 5 (83.3%) | 5 (45%) | 0.304 |
| **Metastatic Disease at Diagnosis** | 1 (14.3%) | 5 (40.0%) | 0.350 |
| **Microvascular Invasion** | 5 (71.1%) | 12 (85.7%) | 0.574 |

**Supplementary Table 4. Age of diagnosis and risk factors between patients that were transplanted and resected.**

|  | **Alive (n=10)** | **Died (n=12)** | **p** |
| --- | --- | --- | --- |
| Infant age (months) | 33.9 (7.9-135.9 | 30.2 (15.2-92.7) | 0.65 |
| **Sex**  Male  Female | 8 (80.0%)  2 (20.0%) | 8 (66.7%)  4 (33.3%) | 0.65 |
| **Race**  Caucasian  Hispanic | 5 (50.0%)  5 (50.0%) | 5 (41.7%)  7 (58.3%) | 0.70  0.70 |
| Premature | 4 (40.0%) | 2 (16.7%) | 0.36 |
| **PRETEXT score***  I  II  III  IV | 0 (0%)  2 (25.0%)  2 (25.0%)  4 (50.0%) | 0 (0%)  0 (0%)  4 (33.3%)  8 (66.7%) | NA  NA  0.69  0.45 |
| Lung metastasis at diagnosis | 5 (50.0%) | 2 (16.7%) | 0.17 |
| V+/P+ | 5 (55.6%) | 5 (41.7%) | 0.67 |
| Microvascular invasion | 8 (88.9%) | 9 (75.0%) | 0.63 |
| **Treatment Scheme**  Medical  Medical and Surgical  None | 1 (10.0%)  9 (90.0%)  0 | 5 (41.7%)  2 (16.6%)  5 (41.7%) | 0.09  0.003  NA |

**Supplementary Table 5. Demographics and initial presentation characteristics of the relapsed patients that lived and those that died.**

**PRETEXT scores were not available for 2 relapsed patients*
